# CK2 targeted RNAi therapeutic delivered via malignant cell-directed tenfibgen nanocapsule: dose and molecular mechanisms of response in xenograft prostate tumors

**DOI:** 10.18632/oncotarget.11442

**Published:** 2016-08-20

**Authors:** Khalil Ahmed, Betsy T. Kren, Md. Joynal Abedin, Rachel I. Vogel, Daniel P. Shaughnessy, Lucas Nacusi, Vicci L. Korman, Yingming Li, Scott M. Dehm, Cheryl L. Zimmerman, Gloria A. Niehans, Gretchen M. Unger, Janeen H. Trembley

**Affiliations:** ^1^ Research Service, Minneapolis VA Health Care System, University of Minnesota, Minneapolis, MN, U.S.A; ^2^ Department of Laboratory Medicine and Pathology, University of Minnesota, Minneapolis, MN, U.S.A; ^3^ Department of Urology, University of Minnesota, Minneapolis, MN, U.S.A; ^4^ Department of Obstretrics, Gynecology and Women's Health, University of Minnesota, Minneapolis, MN, U.S.A; ^5^ Department of Pharmaceutics, University of Minnesota, Minneapolis, MN, U.S.A; ^6^ Masonic Cancer Center, University of Minnesota, Minneapolis, MN, U.S.A; ^7^ GeneSegues Therapeutics, Minnetonka, MN, U.S.A

**Keywords:** prostate, CK2, RNAi, nanoparticle, tumor-specific

## Abstract

CK2, a protein serine/threonine kinase, promotes cell proliferation and suppresses cell death. This essential-for-survival signal demonstrates elevated expression and activity in all cancers examined, and is considered an attractive target for cancer therapy. Here, we present data on the efficacy of a tenfibgen (TBG) coated nanocapsule which delivers its cargo of siRNA (siCK2) or single stranded RNA/DNA oligomers (RNAi-CK2) simultaneously targeting CK2α and α′ catalytic subunits. Intravenous administration of TBG-siCK2 or TBG-RNAi-CK2 resulted in significant xenograft tumor reduction at low doses in PC3-LN4 and 22Rv1 models of prostate cancer. Malignant cell uptake and specificity *in vivo* was verified by FACS analysis and immunofluorescent detection of nanocapsules and PCR detection of released oligomers. Dose response was concordant with CK2αα′ RNA transcript levels and the tumors demonstrated changes in CK2 protein and in markers of proliferation and cell death. Therapeutic response corresponded to expression levels for argonaute and GW proteins, which function in oligomer processing and translational repression. No toxicity was detected in non-tumor tissues or by serum chemistry. Tumor specific delivery of anti-CK2 RNAi via the TBG nanoencapsulation technology warrants further consideration of translational potential.

## INTRODUCTION

The promise of targeted oligomer-based therapeutic drugs for treatment of numerous and varied diseases is enormous, and several recent clinical trials have employed RNAi-based therapy in cancer and other diseases [1]. The ideal goal in cancer therapy is to eliminate the malignant cells rather than stabilize the disease. To that end, the molecular targeting of gene(s) that promote cancer cell growth and survival is an appealing approach. In recent years, interest in RNAi strategies for cancer treatment has continued to gain ground especially since, based on initial phase I clinical trials, it appears that this approach to therapy may be reasonably well tolerated in patients [1]. As discussed, there are several important issues that need to be addressed in developing an RNAi based therapy, including, e.g., the choice of the cancer therapy target and the method of delivering the RNAi therapy to the tumor *in vivo*. Preferably, selection of the target for cancer therapy should include the following considerations. First, it should be dysregulated in all cancer cells; second, there should be no redundancy for its activity; third, it should be essential for cancer cell survival and its downregulation should result in cell death; and fourth, ideally it should be present only in cancer cells.

Protein kinase CK2 (formerly casein kinase II or 2) is a protein serine/threonine (S/T) kinase that is a highly conserved protein across the Eukarya domain. It is involved not only in cell growth and proliferation but is also a potent suppressor of cell death. The latter feature is regarded as a key link of this kinase to the cancer cell phenotype [2]. Importantly, it has been found to be elevated in all cancers examined and its level correlated with prognosis and survival [3–7]. We originally proposed protein kinase CK2 as a target for cancer therapy [8, 9], which meets the first three of the above-mentioned requirements for a cancer therapy target but not the fourth as CK2 is present ubiquitously in all cells. Targeting of CK2 using small molecule inhibitors, peptide inhibitors, and various RNAi strategies leads to cancer cell death in cultured cells and xenograft tumors *in vivo*. CK2 small molecule inhibitors administered orally (CX-4945/Silmitasertib) as well as a peptide-based drug that targets CK2 phosphorylation sites in its substrates and is administered topically or by intralesional injections (CIGB-300) have entered clinical trials for certain cancers, and have not yet progressed past phase I trials as monotherapy or phase I/II in combination therapy (ClinicalTrials.gov Identifier: NCT02128282) [10–12]. In our efforts to target CK2 for cancer therapy, we have embraced the notion of utilizing its molecular downregulation for therapeutic targeting, and because CK2 is ubiquitous and is essential for cell survival, we believe that its targeting warrants delivery of the therapy agent specifically to the cancer cells avoiding the normal cells *in vivo*.

The difficulties of accurate disease cell-specific delivery combined with efficacy have resulted in slow progress; however, encouraging results are emerging. For example, liver targeted delivery of siRNAs using N-acetylgalactosamine (GalNAc) conjugation has shown preliminary efficacy to treat acute hepatic porphyrias [13]. Thus far, the most commonly employed nanoparticle delivery vehicles have predominantly been liposomes. These nanoparticles do not exhibit cancer cell specificity unless an additional ligand is incorporated and their cellular uptake via the coated pit pathway can result in significant loss [14]. To achieve cancer cell-specific targeting of CK2 using an RNAi strategy, we have employed a novel sub-50 nm sized tenfibgen-based delivery vehicle referred to as s50-TBG or TBG nanocapsule, which is designed to restrict delivery of its cargo to cancer cells. The TBG nanocapsule is comprised of a protein shell based on tenfibgen (the carboxy-terminal fibrinogen globe domain of tenascin-C) with the anti-CK2 RNAi oligomer incorporated within this shell. The nanocapsule size is in the 15–30 nm range, and its receptor-mediated entry in cancer cells involves the lipid raft/caveolar pathway where the tenascin receptors are elevated in cancer cells [8, 15].

Here, we report the application of the TBG nanocapsule for delivering double-stranded siRNA or single-stranded RNAi oligomers directed at the two catalytic subunits (CK2α and CK2α′) in two xenograft models of prostate cancer. We describe the tumor response to various doses of the therapeutic nanocapsules, the signaling changes in the tumors, the bioavailability, and the mechanism induced by RNAi delivery at the cellular and molecular levels. We demonstrate the malignant cell specificity of the therapeutic TBG nanocapsules and the lack of any evidence of damage to non-tumor tissues or change in normal serum markers.

## RESULTS

### CK2 expression in multiple models of prostate disease

**Figure 1:**
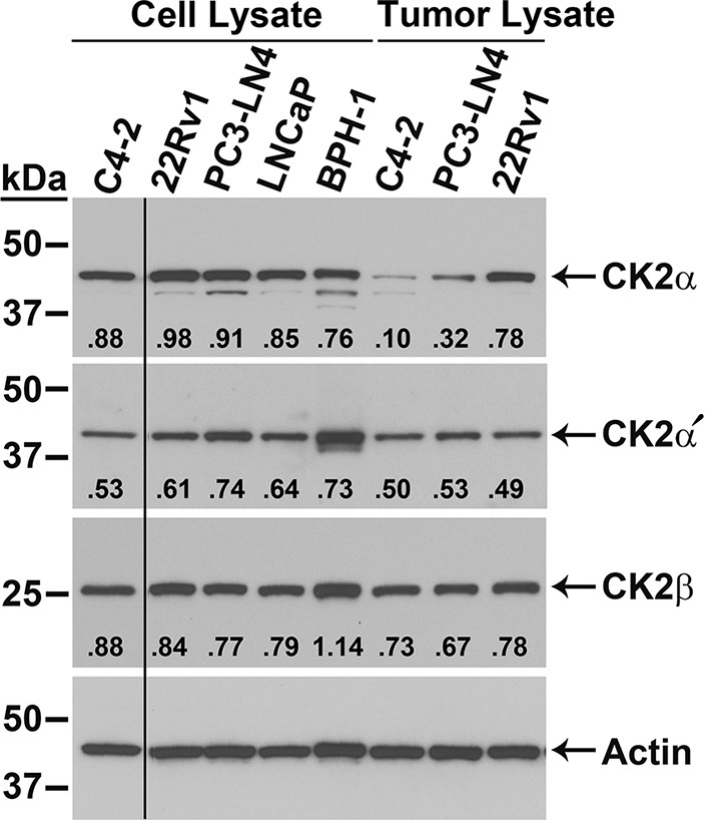
Expression of CK2 protein complex members in malignant and benign prostatic hyperplasia prostate cells Immunoblot analysis of cultured cell and xenograft tumor lysates, as indicated above the blots. Proteins detected are indicated on the right side of the blots. Actin signal was used as the loading control. Antibody sourcing information is listed in Materials and Methods. Band densities relative to actin are listed below each CK2 signal.

The relative mRNA transcript and protein expression levels in multiple prostate cancer cell lines as well as in a benign prostatic hyperplasia cell line were examined, and the results are presented in Figure 1 and Table 1. CK2α and α′ protein levels were similar among the 5 cell lines 22Rv1, PC3-LN4, LNCaP, BPH-1, and C4-2 (Figure 1). For CK2β the highest protein levels were observed in BPH-1 followed by 22Rv1, PC3-LN4, LNCaP and C4-2. We also examined protein expression for cells grown as xenograft tumors. Surprisingly, there was reduced expression for CK2α and CK2α′ in the tumors relative to that in the cultured cells. This could be the result of proteins from mouse stromal cells within the total tissue lysate because the CK2 antibodies used recognize both human and mouse proteins. We have noted in the past that CK2 protein expression levels are frequently lower in stromal cells compared with epithelial cancer cells. CK2β protein expression levels were roughly the same between cultured and tumor cells. We determined mRNA transcript expression levels in cultured and xenograft tumor cells for PC3-LN4, 22Rv1 and C4-2 cell lines using primers specific for human mRNAs in real time quantitative reverse-transcriptase PCR (qRT-PCR) analysis. In contrast to the protein expression data, there was a notable increase in transcript expression relative to HPRT-1 in tumor cells compared to cultured cells for all transcripts except 22Rv1 CK2α′ (Table 1).

**Table 1:** Relative CK2 mRNA expression levels in prostate cancer cell lines and xenograft tumors^a^

	Cell Lines^b^		Xenograft Tumors^c^	
	CK2α	CK2α′	CK2α	CK2α′
PC3-LN4	0.45 ± 0.02	0.22 ± 0.05	1.05 ± 0.09	0.94 ± 0.24
22Rv1	0.83 ± 0.02	0.69 ±0.70	1.99 ± 1.09	0.60 ± 0.13
C4-2	1.61 ± 0.04	0.26 ± 0.03	2.34 ± 0.52	1.02 ± 0.39

a Normalization was performed relative to HPRT-1.

b Data presented as mean ± standard deviation, *n* = 2.

c Data presented as mean ± standard deviation, *n* = 3.

### Tenfibgen nanocapsules for delivery of cargo into tumor cells

For the various tumor growth and cellular response experiments described in the following studies, we employed a tenfibgen (TBG) nanoencapsulation process. The sub-50 nm TBG nanocapsules (hereafter referred to as TBG nanocapsules) contain condensed nucleic acid oligomers, typically display a 15 to 30 nm diameter size, are close to neutral charge, and protect the nucleic acid from degradation [15–17]. TBG nanocapsules are recognized and internalized by malignant tumor cells in the body via the lipid raft/caveolar pathway, and are not taken-up by non-malignant cells such as BPH-1 or tissues such as liver and spleen [15, 17–19]. In the present work, we used two types of anti-CK2 therapeutic nanocapsules in which the oligomers target the same sequence with 100% homology to the CK2α mRNA and 95% homology to the CK2α′ mRNA. The preparation and characterization of double-stranded siCK2 and single-stranded RNAi-CK2 oligomer nanocapsules were described in prior publications [16, 17]. Transmission electron micrographs for representative nanocapsule preparations of the TBG-RNAi-CK2 therapy and TBG-RNAi-F7 control drug used in the dose response studies and the two nanocapsules used in bioavailability studies are provided in Supplementary Figure S1. Detailed characterization of these formulations confirmed their uniform size range, near neutral charge and that the RNAi-CK2 therapy and RNAi-F7 control nanocapsules were very similar in size and charge (Supplementary Table S1).

### Tenfibgen nanocapsule delivery of double and single-stranded anti-CK2 RNAi molecules inhibits prostate xenograft tumor growth

**Figure 2:**
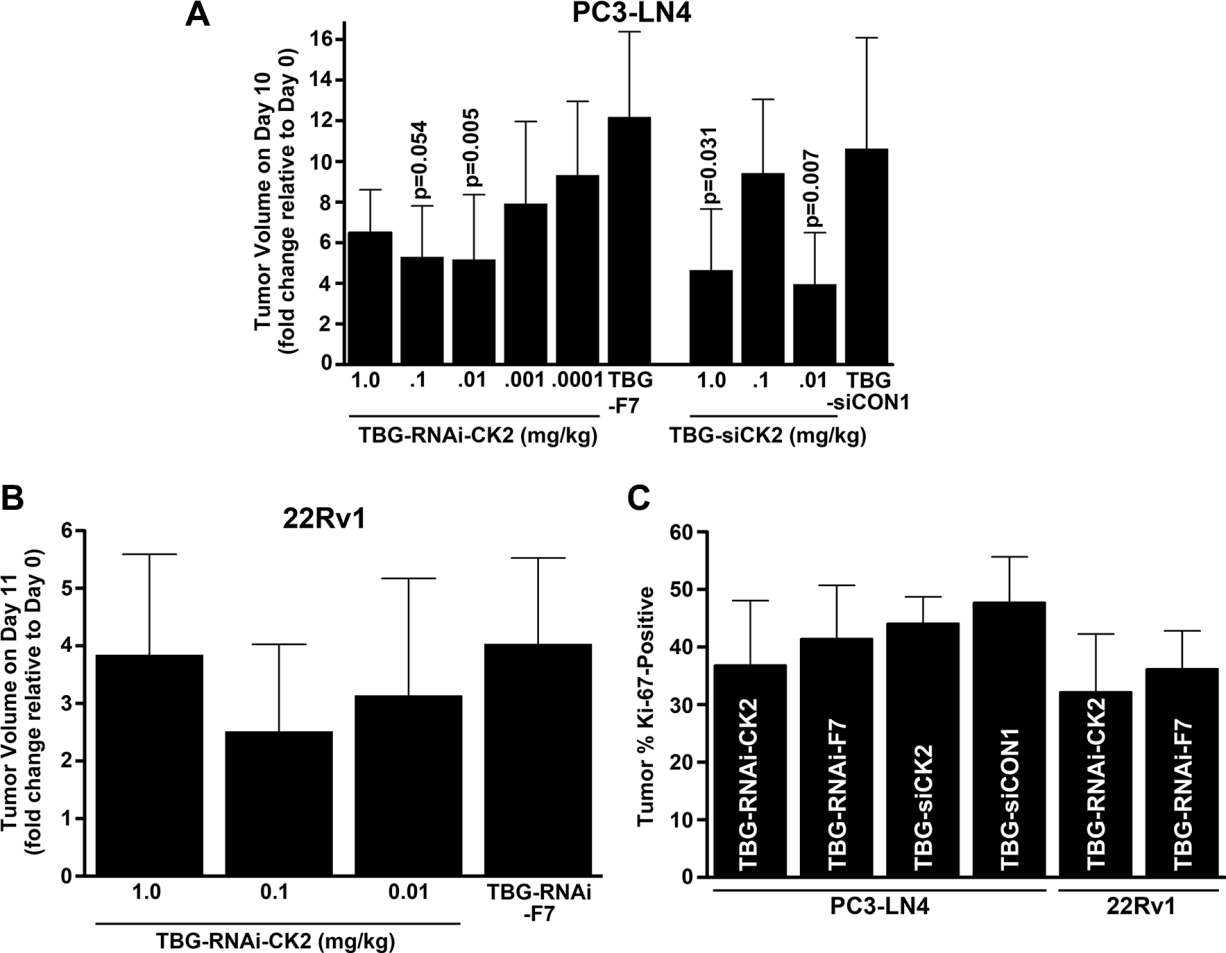
Dose response to TBG-RNAi-CK2 and TBG-siCK2 treatment in PC3-LN4 and 22Rv1 xenograft tumors (**A**) The changes in PC3-LN4 tumor volumes relative to day 0 (day 10/day 0) are shown following drug treatments. Nanocapsule drugs and doses are indicated below the bars. Means ± standard deviations are presented and significance is indicated on the chart. The best response to both TBG-RNAi-CK2 and TBG-siCK2 treatments was observed at 0.01 mg/kg. Group sizes TBG-RNAi-CK2: 1 mg/kg *n* = 3; 0.1 mg/kg *n* = 4; 0.01 mg/kg *n* = 9, 0.001 mg/kg *n* = 8; 0.0001 mg/kg *n* = 8. TBG-RNAi-F7: 0.01 mg/kg *n* = 8. Groups sizes TBG-siCK2: 1 mg/kg *n* = 7; 0.1 mg/kg *n* = 8; 0.01 mg/kg *n* = 9. TBG-siCON1: 1 mg/kg *n* = 8. (**B**) The changes in 22Rv1 tumor volumes relative to day 0 are shown following drug treatments. Nanocapsule drugs and doses are indicated below the bars. Means ± standard deviations are presented. The best response to TBG-RNAi-CK2 treatment was observed at 0.1 mg/kg. Groups sizes TBG-RNAi-CK2: 1 mg/kg *n* = 9; 0.1 mg/kg *n* = 12; 0.01 mg/kg *n* = 8. TBG-RNAi-F7: 0.1 mg/kg *n* = 13. (**C**) The percent of Ki-67 positive cells for PC3-LN4 and 22Rv1 tumors are shown for the best response dose corresponding to 0.01 mg/kg TBG-RNAi-CK2 and TBG siCK2 for PC3-LN4 and 0.1 mg/kg for 22Rv1. The decrease in Ki-67 staining due to anti-CK2 nanocapsule treatment was from 3.6 to 4.6%. Nanocapsule drugs are indicated within the bars. Means ± standard deviations are presented. Sample sizes - PC3-LN4: TBG-RNAi-CK2 *n* = 6; TBG-RNAi-F7 *n* = 8; TBG-siCK2 *n* = 7; TBG-siCON1 *n* = 9. 22Rv1: TBG-RNAi-CK2 *n* =9; TBG-RNAi-F7 *n* = 9.

We carried out acute dose-response experiments with the goal of comparing the efficacy of TBG nanocapsule delivery of single-stranded versus double-stranded anti-CK2 RNAi oligomers. First, we initiated PC3-LN4 flank tumors in male nude mice. Once tumors were established, mice received tail vein injections on days 1, 4 and 7 with various doses of TBG-RNAi-CK2 single stranded oligomer drug or a control drug containing a single-stranded oligomer targeted to mouse factor VII (TBG-F7). Alternatively, mice received various doses of TBG-siCK2 drug or a control drug containing a non-targeting siRNA (TBG-siCON1) [16, 20]. On day 10, 3 days following the final treatment, tumor and other tissues were harvested for analysis. Two dose levels 0.1 and 0.01 mg/kg for TBG-RNAi-CK2 resulted in significantly lower tumor volume relative to day 0 compared to the control group treated with TBG-F7 (*p* = 0.054 and *p* = 0.005, respectively; Figure 2A). Tumor weights were statistically significantly different for TBG-RNAi-CK2 at 0.01 (0.21 ± 0.17 g; *p* = 0.0007; *n* = 9) and 0.1 mg/kg (0.16 ± 0.04 g; *p* = 0.016; *n* = 4) relative to TBG-F7 (0.84 ± 0.54; *n* = 8). For the TBG-siCK2 drug, the dose levels 1.0 and 0.01 mg/kg resulted in significantly slowed tumor growth relative to the control group treated with TBG-siCON1 (*p* = 0.031 and *p* = 0.007, respectively; Figure 2A).

Statistical comparison of the tumor volume changes relative to controls induced by treatment with TBG-RNAi-CK2 vs. TBG-siCK2 was not significant at the best dose of 0.01 mg/kg for each nanocapsule. Given the anomalous dose response characteristics for TBG-siCK2 at 0.1 mg/kg and the similar efficacy of both anti-CK2 oligomer drugs, we chose to move forward with the TBG-RNAi-CK2 drug in further studies. We next performed a similar xenograft flank tumor dose response experiment using the castration resistant prostate cancer model 22Rv1. In this experiment, tumors were initiated in castrated mice. TBG-RNAi-CK2 drug was administered over a dose range from 1 mg/kg down to 0.01 mg/kg by tail vein injection on days 1, 4, 7 and 10. Control TBG-RNAi-F7 drug was administered at 1 mg/kg on the same schedule. On day 11, tumor and other tissues were harvested for analysis. The 22Rv1 xenograft tumors grew much more slowly compared to the PC3-LN4 tumors, and the best reduction in tumor growth was observed with 0.1 mg/kg dose (Figure 2B).

Tumors at the best response dose in the three experiments described above were analyzed for proliferative index as measured by the percentage of cells positive for Ki-67 immunohistochemical signal. There were 3.6 to 4.6% mean percent decreases in Ki-67 positive cells in all treated groups relative to the corresponding control groups, which were not statistically significant (Figure 2C). As discussed subsequently, the reduction in tumor size with little observed difference in the Ki-67 proliferation marker suggests contribution of cell death or other mediators causing reduced tumor volumes.

### Evaluation of tumor response to TBG-RNAi-CK2 over time

**Figure 3:**
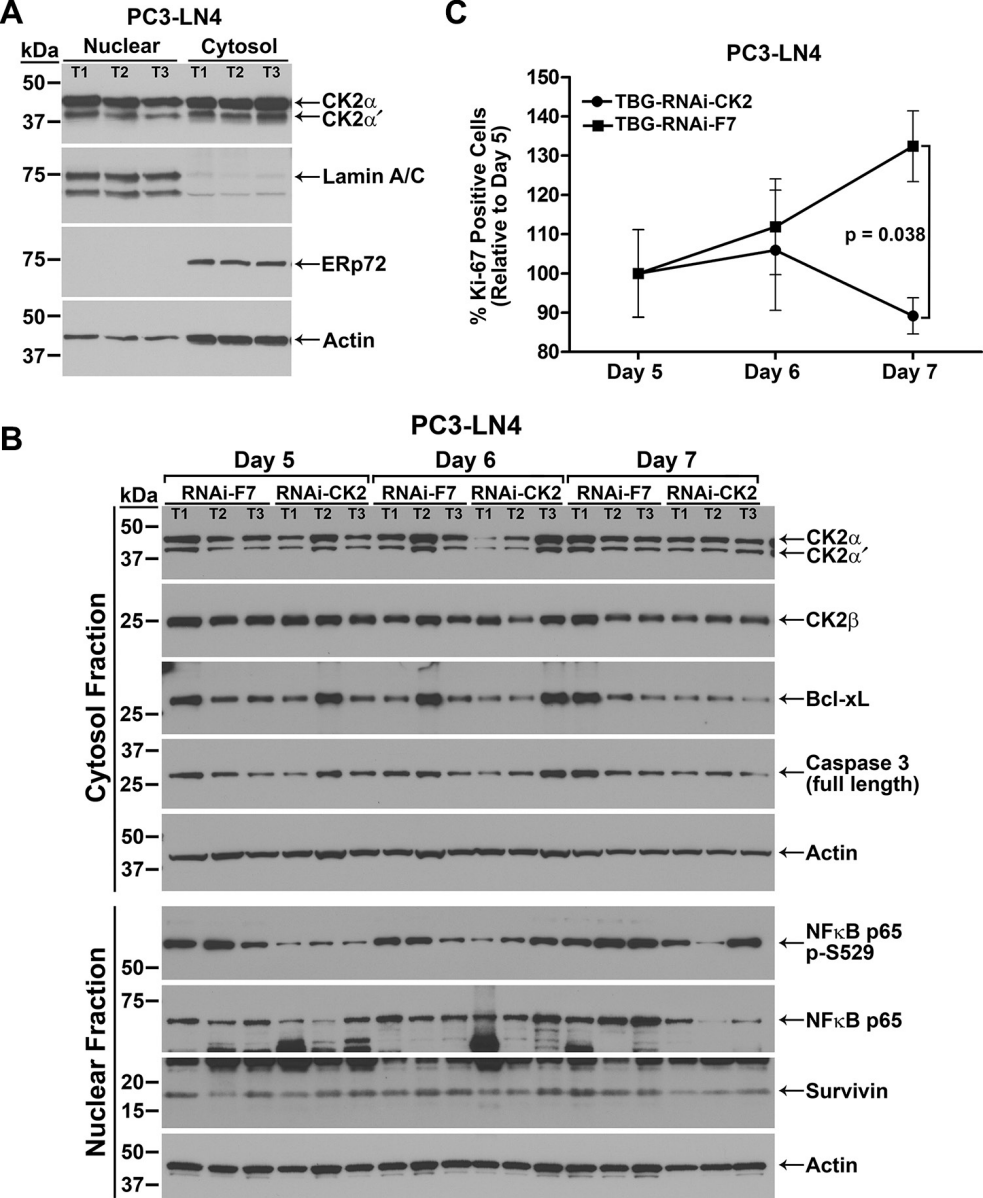
Response over time to TBG-RNAi-CK2 treatment in PC3-LN4 xenograft tumors (**A**) Verification of nuclear and cytosol protein fractionation from tumor tissue is shown by comparative immunoblot analysis of CK2αα′, lamin A/C, ERp72, and actin. T1, T2, and T3 labels indicate different tumors. Antibody sourcing information is listed in Materials and Methods. (**B**) Immunoblot analysis of fractionated PC3-LN4 tumor lysates following intravenous treatments of 0.01 mg/kg TBG-RNAi-CK2 or TBG-RNAi-F7 as indicated above the blots. The signals for three mice per group are shown, the proteins detected are indicated on the right, and the cell fraction and size markers are indicated on the left. Actin signal was used as the loading control. T1, T2, and T3 labels indicate different tumors within each treatment group. Antibody sourcing information is listed in Materials and Methods. (**C**) The percentage of Ki-67-positive tumor cells was determined from Ki-67-stained tumor sections. The percentage of Ki-67-positive cells on each day was expressed relative to day 5 (setting day 5 as 100%) and is shown graphically for each treatment. Mean and standard deviation are shown. Sample sizes: *n* = 6 for all groups except *n* = 7 for TBG-RNAi-F7 day 7.

CK2α and α′ mRNA levels as well as CK2α, α′ and β protein levels were measured in tumor tissue from the dose response studies described above. Although we observed a definite depressed growth response in the anti-CK2 nanocapsule-treated tumors, the CK2 mRNA and protein levels were not notably decreased. We theorized that we must have missed the optimal window of time in which to assess detectable molecular response. Thus, we performed a time course experiment in which tumors were harvested over a 3 day period. The experimental set up to initiate and monitor PC3-LN4 tumor growth was identical to that described for Figure 3 with the exception that we began treatments with slightly larger tumors at an average volume of 145 mm^3^ because we planned to harvest the tumors at earlier time points. Mice were treated on days 1 and 4 with either TBG-RNAi-CK2 or TBG-F7 at a dose of 0.01 mg/kg. Tumors were collected on days 5, 6 and 7 from 6 mice per treatment group on each day.

We first performed immunoblot analysis of fractionated tumor tissue. The data shown in Figure 3A demonstrates the efficacy of the nuclear and cytosolic fractionation procedure using the luminal endoplasmic reticulum protein ERp72 as a cytosol marker and A-type lamin A and C as nuclear markers. The data also show that actin is detected in both the cytosol and nucleus using a broad range antibody, thus we used actin to normalize all data. Protein expression levels were analyzed by immunoblot using fractionated lysates for all TBG-RNAi-CK2 and TBG-F7 treated tumors. The data presented in Table 2 for TBG-RNAi-CK2 treatment protein levels represents the 3 best responding tumors based on the change in tumor volume on the collection day relative to day 0, the day before treatment started. We chose this approach to data analysis as we wanted to determine the molecular changes in responding tumors. The data for protein levels in TBG-RNAi-CK2 tumors were expressed relative to all 6 control TBG-F7 tumors from the same day. The data shown in Figure 3B, though generally representative, do not represent the best responders in all lanes for the TBG-RNAi-CK2 groups.

**Table 2:** Changes in protein expression over time in TBG-RNAi-CK2 treated xenograft tumors

Protein Detected	Day 5		Day 6		Day 7	
	Mean ± SD	*p*-value	Mean ± SD	*p*-value	Mean ± SD	*p*-value
CK2α - cyto	0.54 ± 0.21	0.04	0.73 ± 0.59	0.51	0.90 ± 0.09	0.25
CK2α – nuc	0.75 ± 0.17	0.11	1.23 ± 0.71	0.64	1.09 ± 0.23	0.58
CK2α′ - cyto	0.52 ± 0.31	0.10	0.66 ± 0.45	0.32	0.92 ± 0.20	0.59
CK2α′ - nuc	0.56 ± 0.24	0.11	1.49 ± 1.12	0.53	0.93 ± 0.42	0.80
CK2β – cyto	0.73 ± 0.24	0.20	0.97 ± 0.30	0.90	1.00 ± 0.21	0.99
CK2β - nuc	1.25 ± 0.66	0.59	1.10 ± 0.16	0.54	0.92 ± 0.16	0.51
NFκB p65 – cyto	0.90 ± 0.07	0.47	0.90 ± 0.34	0.67	0.79 ± 0.10	0.05
NFκB p65 – nuc	1.07 ± 0.04	0.84	1.28 ± 0.41	0.39	0.33 ± 0.32	0.05
NFκB p65 p-S529 - nuc	0.38 ± 0.24	0.11	1.21 ± 1.16	0.79	0.52 ± 0.31	0.10
Casp 3FL – cyto	0.68 ± 0.22	0.13	0.80 ± 0.33	0.40	0.64 ± 0.20	0.06
Bcl-xL - cyto	0.60 ± 0.04	0.07	0.84 ± 0.64	0.72	0.43 ± 0.14	0.01
Survivin - nuc	0.91 ± 0.58	0.82	1.01 ± 0.37	0.98	0.41 ± 0.14	0.008

Normalization was performed relative to actin; all TBG-F7 mean quantitation values set as value of 1.

Data presented as mean ± standard deviation (SD), *n* = 3 for TBG-RNAi-CK2, *n* = 6 for TBG-F7 days 5 & 6, *n* = 7 for TBG-F7 day 7.

Casp 3^FL^ = full length caspase 3 or pro-caspase 3.

The nuclear and cytosolic tumor lysate fractions were analyzed by immunoblot for the CK2 holoenzyme components. Significant loss of CK2α and α′ was observed in the cytosolic fraction on day 5 (*p* = 0.04), with increasing amounts of protein detected on days 6 and 7 (Table 2 and Figure 3B). Nuclear CK2α and α′ proteins showed a slightly lesser response and protein levels recovered faster. CK2β protein was moderately decreased in the cytosolic fraction on day 5. NFκB p65 is phosphorylated by CK2 on serine 529 (p-S529) [21]. Biphasic loss of nuclear NFκB p65 p-S529 phosphorylation was seen on days 5 and 7; whereas significant loss of total nuclear NFκB p65 protein was observed on day 7 (*p* = 0.05; Table 2). Statistically significant loss of total cytosolic NFκB p65 protein was also observed, though to a much lesser extent than that of nuclear, on day 7 (*p* = 0.05). Biphasic reduction in Bcl-xL and full length pro-caspase 3 also occurred on days 5 and 7, with more significant loss on day 7 (*p* = 0.01 and *p* = 0.06, respectively). Finally, there was a significant downregulation of survivin protein on day 7 (*p* = 0.008; Figure 3B and Table 2).

Because the greatest CK2 protein loss was observed on day 5, we planned to use tumors from this time point to perform qPCR. Unfortunately, there was insufficient tumor material remaining from the best responding tumors (according to tumor volume change) for further analysis, thus the mRNA analysis represents the remaining 3 tumors for TBG-RNAi-CK2. All mRNA levels were expressed relative to HPRT-1 levels which were set as 1. CK2α and α′ mRNA levels were 0.82 ± 0.32 and 0.77 ± 0.11, respectively, in TBG-RNAi-CK2 treated tumors relative to 0.98 ± 0.13 and 0.83 ± 0.15, respectively, in TBG-F7 treated tumors.

The percent Ki-67 positive cells was determined in all TBG-RNAi-CK2 and TBG-F7 tumors for each day of the time course. The TBG-RNAi-CK2 treated tumors demonstrated a sharp decline in proliferative index from day 6 to day 7, whereas the TBG-F7 treated tumors continued to show increased proliferation from day 5 through day 7 (Figure 3C). The test for interaction effect between treatment and day in TBG-RNAi-CK2 tumors, which dropped in proliferative index by 15% from Day 5 to Day 7, was statistically significant relative to TBG-F7 tumors, which increased in proliferative index by 32% (*p* = 0.038).

### Cellular mechanisms of response to nanocapsule-delivered RNAi drug

The nanocapsule dose response difference between the two prostate cancer models impelled us to investigate possible model characteristics influencing treatment response. In addition, the PCR results from the time course experiments demonstrated a discrepancy between CK2 protein and mRNA levels, suggesting that additional molecular mechanisms are involved in the CK2 protein loss observed.

**Figure 4:**
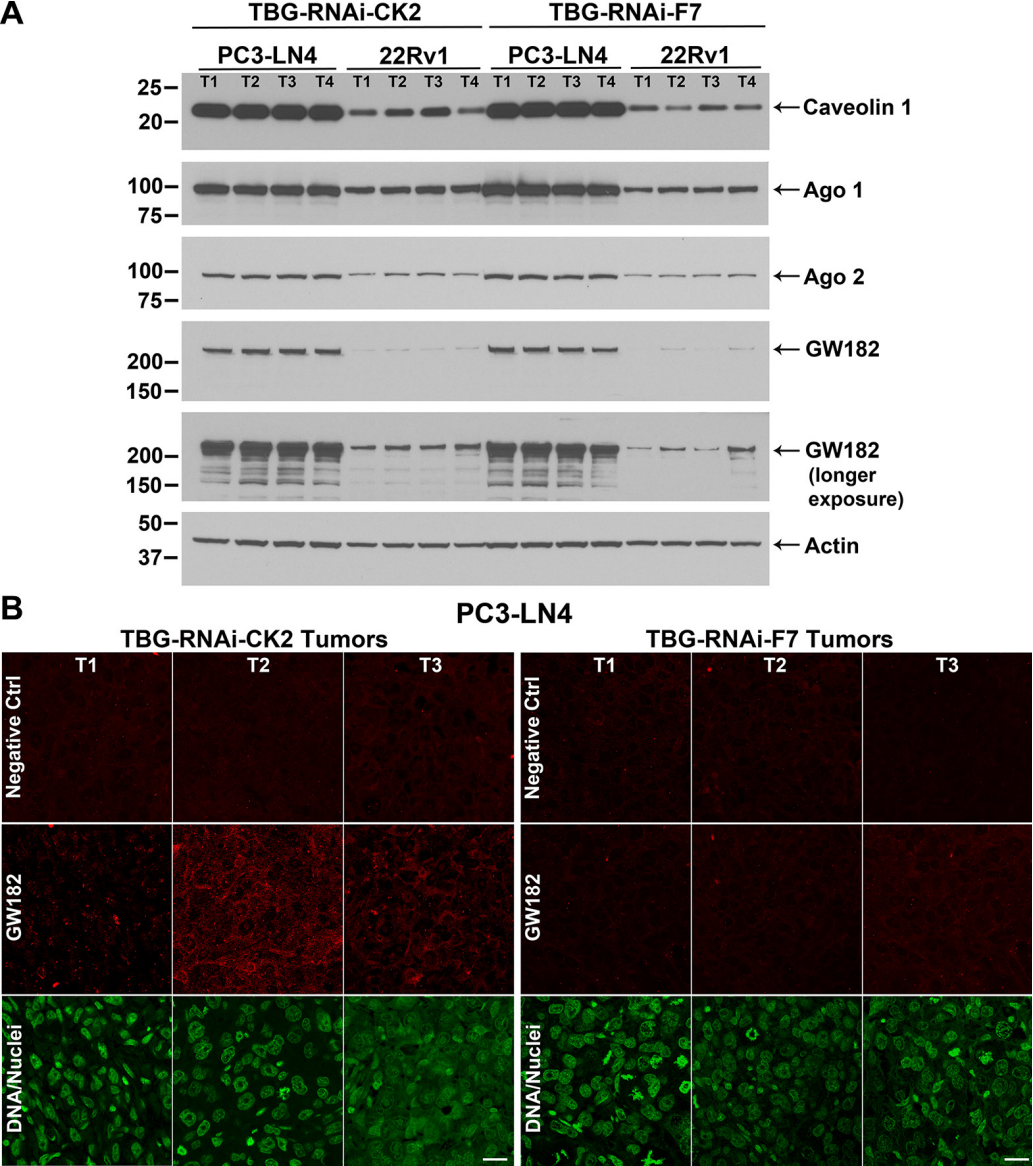
Cellular expression of TBG nanocapsule uptake and RNAi-CK2 oligomer processing markers in xenograft tumors (**A**) Expression levels for key nanocapsule entry and oligomer processing proteins were detected by immunoblot in PC3-LN4 and 22Rv1 cytosolic tumor lysates from the dose response studies. The signals for four mice per group are shown, the proteins detected are indicated on the right, and the size markers are indicated on the left. Two exposures are provided for GW182 in order to show detectable signals in linear range in all lanes for both PC3-LN4 and 22Rv1 tumor lysates. T1, T2, T3, and T4 labels indicate different tumors within the treatment and xenograft model groups. Antibody sourcing information is listed in Materials and Methods. Actin signal was used as the loading control. (**B**) Indirect immunofluorescence detection of GW182 proteins and GW bodies in PC3-LN4 tumors. Results from 3 mice treated with TNG-RNAi-CK2 and 3 mice treated with TBG-RNAi-F7 are shown. T1, T2, and T3 labels indicate different tumors. Antibody sourcing information is listed in Materials and Methods. Nuclei were counterstained with Sytox^®^ Green. Scale bar represents 20 μm.

We first investigated if levels for caveolin 1, a protein involved with TBG nanocapsule entry by the caveolar or lipid-raft type mechanism [15] in tumor cells, were differentially expressed. We compared levels in anti-CK2- *vs*. control-treated cytosolic tumor lysates from all of the dose response experiments. Caveolin 1 expression was much higher in PC3-LN4 than in 22Rv1 cells (Figure 4A). Argonaute 1 and 2 (Ago 1 and Ago 2) are proteins which associate with small RNAs, such as miRNAs and siRNAs, and function in RNA interference pathways to reduce protein expression through mRNA loss or through repression of protein translation [22]. GW182 (TNRC6A) proteins act as adaptor proteins, coordinating with Ago proteins to achieve gene silencing [22]. We found higher Ago 1, Ago 2 and especially GW182 protein signals in PC3-LN4 tumor lysates compared to those from 22Rv1 xenografts (Figure 4A). Although phosphorylation of Ago 2 at serine 387 has been shown to facilitate interaction with GW182 and localization to GW bodies, and thus downregulate its cleavage function, we did not detect any notable change in Ago 2 p-S387 signal (data not shown) [23]. There were no notable differences between TBG-siCK2 and TBG-siCON1 tumor lysates for the Ago or GW proteins (data not shown).

Next we examined GW182 protein localization by indirect immunofluorescence in TBG-RNAi-CK2 and TBG-F7 treated PC3-LN4 tumors at day 5 from the time course experiment. We observed a notable increase in GW182 diffuse and punctate signals in TBG-RNAi-CK2 treated tumors compared to the TBG-F7 treated tumors (Figure 4B). Because prostate cancer cells do not express Factor VII, there is no mRNA target for the murine TBG-RNAi-F7 oligomer, and therefore no induction of GW bodies would be anticipated in the control tumors [24].

### Nanocapsule uptake and oligomer bioavailability *in vivo*

**Figure 5:**
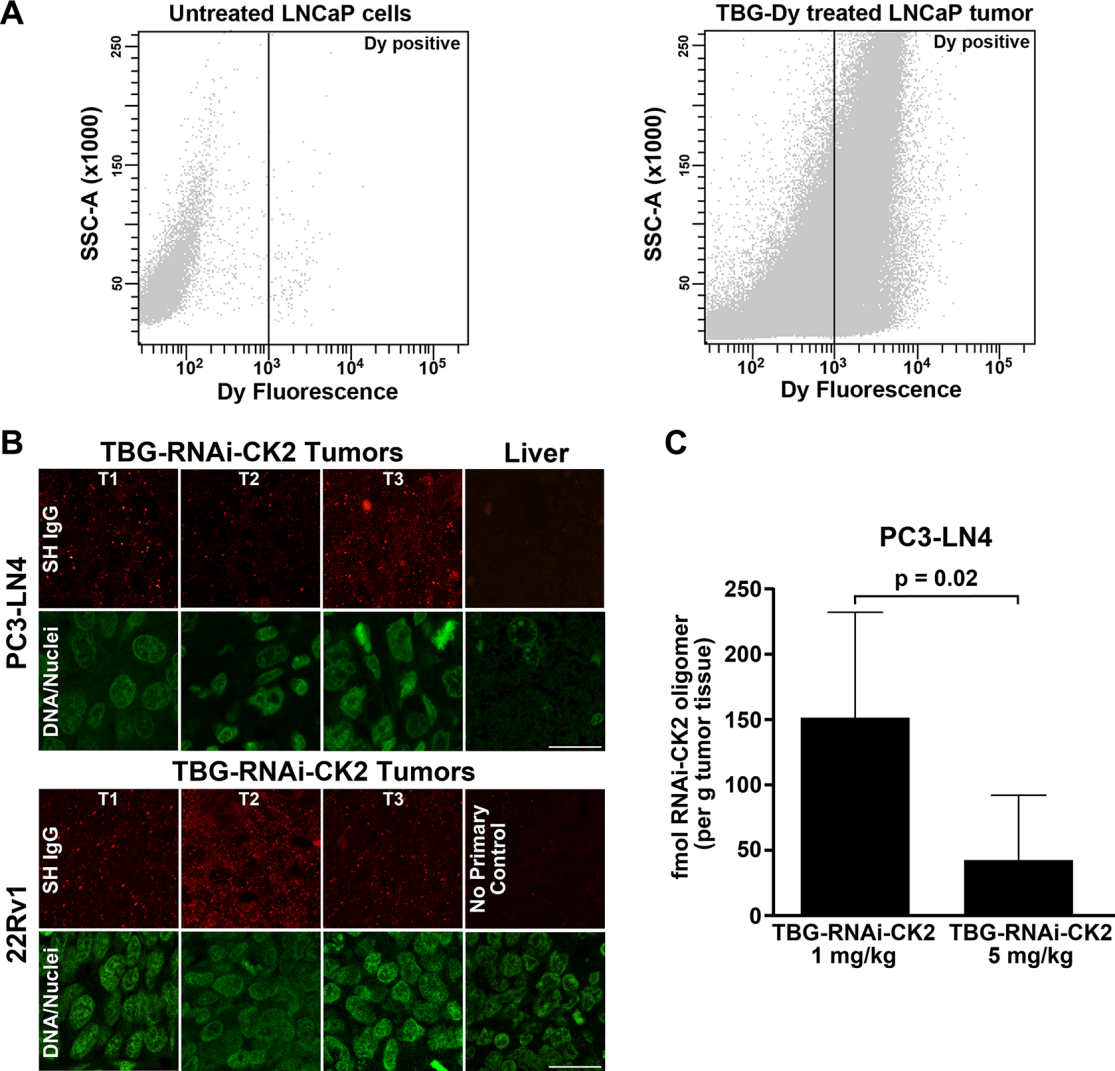
Efficiency and verification of TBG nanocapsule delivery and detection of RNAi-CK2 oligomer released in xenograft tumors (**A**) FACS analysis of untreated LNCaP cells (left panel) and TBG-Dy treated LNCaP xenograft tumor cells (right panel). The position of the gate set to define Dy-positive cells is shown as a black line with Dy-positive events to the right of the line. (**B**) Tumor and liver sections from mice treated with TBG-RNAi-CK2 were collect 24 h post treatment (Day 5 for PC3-LN4; Day 11 for 22Rv1) and subjected to indirect immunofluorescence analysis for Syrian hamster IgG contained in the nanocapsules. Nuclei were counterstained with Sytox^®^ Green. T1, T2, and T3 labels indicate different tumors. Scale bar is 20 μm. (**C**) The graph depicts the mean fmols of RNAi-CK2 recovered and detected by q-SL-RT-PCR in PC3-LN4 xenograft tumors. Mice were treated once by tail vein injection at the dose indicated under each bar. Means (*n* = 6 per group) are presented and error bars represent standard deviation. The *p*-value is indicated on the graph.

We were interested in quantifying the number of tumor cells which take up nanocapsule when mice carrying prostate tumors are systemically injected. In order to accomplish this, TBG nanocapsules containing a dysprosium (Dy) dextran cargo were used because Dy is a fluorescent lanthanide element which is detectable by fluorescence activated cell sorting (FACS) analysis. Mice carrying LNCaP orthotopic xenograft tumors were injected via tail vein or intraperitoneal routes with TBG-Dy nanocapsules. The tumors were collected after a 20 h interval and the cells were dissociated. Using untreated LNCaP cells to set the gate (Figure 5A left panel), FACS analysis for Dy signal was performed on the 3 Dy tumors (Figure 5A right panel and Table 3). After just one injection, 46.3 ± 0.4% of the tumor cells were Dy-positive. Additionally, the amount of TBG-Dy nanocapsule detected in the tumor cells was equivalent for the tail vein and intraperitoneal injection routes.

**Table 3:** FACS analysis of TBG-Dy uptake in orthotopic tumors

LNCaP Cells/Tumor	Treatment	Injection	% Dy Positive
Cells	Untreated	N/A	1.5
Tumor 1	TBG-Dy	iv	46.0
Tumor 2	TBG-Dy	iv	46.3
Tumor 3	TBG-Dy	ip	46.7

Mice received 200 nmol/kg TBG-Dy by intraperitoneal (ip) or tail vein (iv) injection 20 h prior to tumor excision and processing.

TBG nanocapsule uptake was evaluated *in vivo* by indirect immunofluorescence detection of the nanocapsules. Specific detection of the nanocapsules in the tissues is possible using an anti-Syrian hamster IgG antibody due to incorporation of the hamster IgG into the TBG protein coat during formulation. Tumor sections were processed for hamster IgG signal from PC3-LN4 and 22Rv1 xenografts 24 h after treatment with TBG-RNAi-CK2. The results showed that nanocapsules were detected to fairly equal intensities of signal in both type of xenograft tumors (Figure 5B). No nanocapsule signal was detected in liver (far right section in upper panel of Figure 5B).

A further xenograft tumor experiment was performed to address other important conceptual questions about our nanocapsule delivery technology. First, we wished to formally test that delivery of RNAi oligomers to tumors was accomplished specifically as a consequence of TBG nanoencapsulation of the oligomer cargo, and not as non-specific association of the oligomer with the protein nanocapsule. Second, we wished to detect released RNAi oligomer drug within tumor cells. The third goal was to determine whether any oligomer could be detected in the potential off-target organs such as liver and spleen. To accomplish these goals, we used the RNAi-CK2 oligomer with a standard 3′-OH chemistry so we could perform quantitative stem-loop RT-PCR (q-SL-PCR) on RNA purified from tissues. Mice carrying PC3-LN4 xenograft flank tumors (at least 250 mm^3^) were injected with 5 mg/kg TBG-RNAi-CK2, or 1 mg/kg TBG-RNAi-CK2, or 5 mg/kg “naked” RNAi-CK2 oligomer in combination with TBG-erythritol nanocapsules at the same particle number as the TBG-RNAi-CK2. Tumors were collected exactly 24 h after treatment. To ensure that oligomer detected in TBG-RNAi-CK2 treated tumors was released *in vivo* and not as a consequence of tissue processing, equal amounts of untreated PC3-LN4 tumor lysate were either spiked with TBG-RNAi-CK2 nanocapsule or naked RNAi-CK2 or vehicle and processed for RNA purification. Following q-SL-RT-PCR, the RNAi-CK2 oligomer was only detected in the tumor lysate spiked with naked oligomer (data not shown).

The RNAi-CK2 oligomer was detected by q-SL-RT-PCR in all 6 tumors from mice injected with 1 mg/kg TBG-RNAi-CK2, ranging in total recovery per tumor from 29 to 119 fmol. The RNAi-CK2 oligomer was detected in only 3 of the 6 tumors from mice injected with 5 mg/kg TBG-RNAi-CK2, and the amounts ranged from 18 to 63 fmol (Table 4). No RNAi-CK2 oligomer was detected in the tumors from any of the “naked” RNAi-CK2/TBG-erythritol injected mice. The difference in mean recovered oligomer drug per gram of tumor between 1 mg/kg and 5 mg/kg TBG-RNAi-CK2 treatments was significant (*p* = 0.02, Figure 5C). Liver and spleen from 3 mice per group were also processed for detection of RNAi-CK2 oligomer. RNAi-CK2 at 442.5 fmol (corresponding to 1042 fmol if adjusted for spiked oligomer recovery in tumor tissue) was detected in 1 liver from a 5 mg/kg “naked” oligomer TBG-erythritol injected mouse. RNAi-CK2 at 4.7 fmol (corresponding to 11 fmol if adjusted for spiked oligomer recovery in tumor tissue) was detected in 1 spleen from a 5 mg/kg TBG-RNAi-CK2 injected mouse.

**Table 4:** Bioavailable oligomer detected in tumors

Treatment group	Tumor Mass (g)	Total Oligomer (fmol)	Oligomer per g Tumor (fmol)
TBG-RNAi-CK2 1 mg/kg	0.60	38.6	64.7
	0.58	76.3	131.4
	0.39	61.9	160.4
	0.43	80.5	188.9
	0.42	118.8	284.9
	0.37	29.0	78.9
TBG-RNAi-CK2 5 mg/kg	0.32	17.8	56.6
	0.26	0	0
	0.25	22.4	90.3
	0.58	63.0	108.7
	0.51	0	0
	0.90	0	0

### Systemic impact following intravenous nanocapsule treatment

Evaluation for tissue damage by blood serum chemistry and tissue morphology in TBG nanocapsule treated mice was undertaken. Blood serum chemistry values were determined for TBG-RNAi-CK2 and TBG-F7 treated mice in the 22Rv1 dose response experiment, using animals that received the highest dose amount in each group. Results shown in Table 5 for markers of liver and kidney function indicated no perturbations of organ function. Further, liver, spleen and kidney tissue sections from TBG-RNAi-CK2, TBG-F7, TBG-siCK2, and TBG-siCON1 treated mice in the various dose response experiments were stained using hematoxylin and eosin and examined by a pathologist. No tissue damage was observed (data not shown). Finally, mice in all treatment groups demonstrated weight gain throughout the studies.

**Table 5:** Blood serum chemistry values in tumor-bearing TBG nanocapsule treated mice^a^

Measure	*n*	TBG-F7 Mean ± SD^b^	*n*	TBG-RNAi-CK2 Mean ± SD	*p*-value^c^
Blood Urea Nitrogen (BUN)	6	22.0 ± 5.3 mg/dL	6	20.5 ± 5.3 mg/dL	0.63
Creatinine	6	0.20 ± 0 mg/dL	6	0.20 ± 0 mg/dL	−
Total Serum Protein (TP)	6	4.55 ± 0.15 g/dL	6	4.60 ± 0.20 g/dL	0.64
Alanine Aminotransferase (ALT)	6	19.8 ± 1.5 U/L	6	23.3 ± 4.1 U/L	0.10
Aspartate Aminotransferase (AST)	5	69.6 ± 11.5 U/L	6	86.3 ± 13.8 U/L	0.06

a Serum was collected 24 h after fourth nanocapsule injection.

b SD = standard deviation.

c Comparison of TBG-RNAi-CK2 vs. TBG-RNAi-F7 by *t*-test assuming unequal variances.

## DISCUSSION

The PC3-LN4 xenograft tumors demonstrated the best dose response to CK2 downregulation at 0.01 mg/kg of single-stranded (RNAi-CK2) and double-stranded (siCK2) oligomer delivery. The 22Rv1 xenograft model responded best at 0.1 mg/kg of TBG-RNAi-CK2; PC3-LN4 tumors also demonstrated good response at this dose level. While treatment with TBG-siCK2 demonstrated good response at 1 and 0.01 mg/kg, there was lack of response in the interval dose at 0.1 mg/kg, noted with two separate lots of nanocapsule and two independent dose response experiments. Thus, while elucidating this anomalous tumor response to TBG-siCK2 treatment is a future goal, we chose to focus on using the single-stranded RNAi-CK2 oligomer for the various studies.

Even with dramatic changes in tumor volumes and weights, molecular and cellular response measures were unremarkable at 3 days following final TBG-RNAiCK2 treatment suggesting we may have missed the optimal sampling times to examine the cellular pathways involved. Thus, we examined response markers over a 3 day time course following 2 treatments and collecting tumors on days 5, 6 and 7 as this is the time frame when we detect that the tumor volume changes begin to be notably different between RNAi-CK2 and RNAi-F7 treatments. An overall loss of CK2 protein expression was observed 24 h post TBG-RNAi-CK2 treatment on day 5. Coincident with loss of CK2 on day 5, decreased expression levels were observed for Bcl-xL, full length caspase 3, and NFκB p65 p-S529. These early changes in cell survival and proliferation signals might reflect an acute response to decreased CK2 activity. We have observed in cultured prostate cancer cells that abrupt loss of CK2 activity for just 6 hours caused significantly reduced clonal cell survival, in part due to reduction in mitochondrial membrane potential [25]. The most profound induction of death signaling in TBG-RNAi-CK2 treated tumors was noted 72 h post treatment, involving loss of nuclear NFκB p65, full length caspase 3, Bcl-xL and survivin. These changes indicate induction of cell death at a point in time when CK2 protein levels appear to have recovered. Complex modes of CK2 regulation of cell death exist and might involve not only its relatively modest downregulation or inhibition, but also its intracellular and intracompartmental redistribution, as has been observed in cultured cells and xenograft tumors [26–28]. We theorize that a biphasic tumor cell response is occurring. In the first phase, a population of tumor cells which received TBG-RNAi-CK2 nanocapsule downregulated CK2 expression to an extent that, among other insults, caused loss of mitochondrial membrane potential and induced cell death. In the second phase, a population of tumor cells which might have downregulated CK2 to a lesser extent or potentially changed its intracellular distribution experienced a cumulative imbalance of CK2-mediated survival signaling leading to eventual death. This second phase of cell death induction is exemplified by reduced nuclear survivin and cytosolic Bcl-xL, key survival proteins in cancer [29, 30]. Our data suggests that apoptosis represents a portion of the tumor cell death induced by TBG-RNAi-CK2 treatment in PC3-LN4 xenografts, a model which completely lacks PTEN. In contrast to our method of targeting CK2 in which the protein itself is lost, use of the CK2 small molecule inhibitor CX-4945 to treat prostate tumors in prostate-specific PTEN knockout mice resulted in induction of senescence rather than cell death [31].

We have previously reported effects of CK2 downregulation both *in vivo* and *in vitro* on the NFκB signaling pathway in prostate and in head and neck squamous cell carcinoma [15, 17, 19, 32]. CK2 loss had the largest impact on nuclear NFκB p65, which is consistent with a report that CK2 interacts with NFκB p65 to a greater extent in the nucleus vs. the cytosol [33]. Furthermore, we have observed in related work that high levels of CK2α protein expression accounted for increased levels of nuclear NFκB p65 protein levels in prostate cancer tissues relative to BPH tissues [34]. Therefore, it is possible that loss of CK2 protein levels in TBG-RNAi-CK2 treated tumors had an early effect on phosphorylation of nuclear NFκB p65 p-S529, thus decreasing NFκB activity, on day 5 and a later effect on total nuclear NFκB p65 levels on day 7.

The dose response results indicate that PC3-LN4 tumors respond better overall and at lower dosages than 22Rv1 tumors. One reason for this difference is that CK2α mRNA and protein levels in 22Rv1 tumors are two-fold higher than in PC3-LN4 tumors (Figure 1 and Table 1). Other likely factors influencing the more sensitive response in PC3-LN4 tumors are the notably higher expression levels of proteins which mediate drug processing within the cells, such as Ago 1, Ago 2, and GW182. Our data showed roughly equivalent detection of nanocapsule drug in both tumor types, suggesting that, although caveolin 1 protein levels were higher in PC3-LN4 xenografts, there was sufficient caveolin 1 in 22Rv1 xenografts for similar levels of drug entry.

We have published data showing that a modest amount of RISC-like cleavage of the CK2α mRNA occurs at the appropriate oligomer-targeted site in the transcript following TBG-siCK2 and TBG-RNAi-CK2 treatment of breast and prostate xenograft tumors, respectively [16, 17]. In the experiments reported here, we observed moderately decreased CK2α and α′ mRNA expression in the less well-responding tumors on day 5 corresponding to 24 h post-treatment. This modest loss of CK2 transcripts, in part, reflects the fact that we did not have tumor tissue remaining from the best responders for the mRNA measurements. It is also possible we missed the window of time in which to capture significant CK2 mRNA transcript loss, which would presumably be prior to 24 h post TBG-RNAi-CK2 treatment. Alternatively, our data also suggest that more than one mechanism is employed by tumor cells to decrease CK2 protein expression in response to the nanocapsule-mediated uptake of RNAi oligomers.

In the present work, we have endeavored to identify the underlying mechanism of gene silencing by the TBG nanocapsule-delivered oligomers. Available published data suggest that gene silencing is accomplished through either translational repression or by cleavage- or deadenylation/decapping-induced transcript loss. The details of how endogenous miRNAs repress translation are not yet fully understood, but it appears that miRNAs inhibit translation through the cap-dependent initiation step [22]. Additionally, a recent study demonstrated that phosphorothioate locked nucleic acid single stranded gapmer oligomers localize to GW bodies in a pattern identical to that of siRNAs [35]. While translational repression is currently thought to account for no more than 24% of gene silencing in cultured cells [36], it is possible that introduction of an exogenous RNAi oligomer targeting an intra-open reading frame site into tumor cells in an *in vivo* environment triggers translational repression as a more chronic mechanism over transcript degradation (which may be an acute response) [24]. The single-stranded RNAi-CK2 oligomer employed by us may be inducing miRNA-like translational repression mechanisms in the tumor cells consistent with the robust expression of Ago1 relative to Ago2 in the xenografts [37]. Our observations of increased punctate GW body detection in TBG-RNAi-CK2 treated tumors suggests that translational repression via sequestration of the CK2αα′ mRNAs is, at least partially, responsible for decreased CK2α and α′ protein levels in the tumors.

In the bioavailability experiment, we could not detect RNAi-CK2 oligomer in tumors from mice injected with “naked” oligomer in combination with TBG-erythritol nanocapsules. The liver is a well-established organ for oligomer accumulation after systemic introduction in a non-targeted manner, and RNAi-CK2 oligomer was detected in 1 mouse liver after introduction of “naked” oligomer [38]. These data support our assertion that the oligomer is, in fact, encapsulated within the TBG for malignant cell-specific delivery, and is not associated non-specifically with nanocapsules. The data also emphasize the vast improvement in oligomer delivery afforded by TBG nanoencapsulation. Interestingly, the amounts of oligomer we detected in the prostate xenograft tumors at a dose of 1 mg/kg TBG-RNAi-CK2 (range 0.4 to 1.8 ng/g tissue) were very similar to detection of VEGF and KSP siRNAs after a dose of 1 mg/kg ALN-VSP (range 0.4 to 9.8 ng/g tissue) reported by others [39]. Moreover, the amount of oligomer released within the 5 mg/kg TBG-RNAi-CK2 tumors was less than that in the 1 mg/kg dosed tumors, and a similar inverse relationship between amount of oligomer drug injected and the amount recovered in tumor biopsies, comparing 1 mg/kg and 0.4 mg/kg, was reported by Tabernero and colleagues [39]. Thus, although the ALN-VSP drug is a non-targeted lipid nanoparticle and TBG-RNAi-CK2 is a tumor-targeted nanocapsule, some of their tumor cell delivery characteristics are similar.

In conclusion, by employing two models of xenograft prostate cancer we have examined the activity, biodistribution and possible mechanism of function of the TBG nanocapsule carrying anti-CK2 RNAi oligomers. We have established that the RNAi-CK2 resides within the TBG nanocapsule and that it delivers the RNAi-CK2 into the tumor cells and avoids perturbation of normal cell function. Our results demonstrate that the delivery of the RNAi-CK2 cargo to tumor cells produced effects on tumor reduction, with the sensitivity of the response being modulated by target abundance and expression of proteins associated with the pathways involved in RNA silencing. Moreover, the negative impact on tumor growth caused by RNAi-CK2 treatment appears to result from induction of cell death rather than simply decreased proliferation. In other work, we have demonstrated delivery of TBG nanocapsules to metastatic sites, including lymph node and bone tumors in prostate cancer and spleen tumors in head and neck squamous cell carcinoma [15, 17, 40]. Moreover, we have observed reduction in the presence and size of metastatic tumors as well as loss of CK2-related signaling in metastases in response to TBG-RNAi-CK2 and TBG-AS-CK2 treatment [15, 17]. Taken together, these observations support the possible utility of this cancer-targeted therapy for primary and metastatic tumors.

## MATERIALS AND METHODS

### Cell lines and culture

PC3-LN4, C4-2 and BPH-1 cells were obtained and maintained as described, with the exception that fetal bovine serum (FBS) was not heat-inactivated [19]. 22Rv1 (CRL-2505) and LNCaP (CRL-1740) cells were obtained from ATCC (Manassas, VA, USA) and were maintained in RPMI-1640 with 10% FBS and 1% penicillin-streptomycin. Cells were grown in an incubator at 37°C with 5% CO_2_. All cells had undetectable levels of mycoplasma when thawed, and were maintained in culture for up to 3 months.

### Cell and tumor lysate preparation and immunoblot analysis

Cell pellets from cultured cells were processed in radioimmunoprecipitation assay (RIPA) buffer as described [41]. For tumor whole cell lysates, approximately 0.05 g of frozen tumor tissue was minced and homogenized on ice in Buffer A1 (10 mM Tris pH 7.4, 5 mM MgCl_2_, 25 mM KCl, 1 mM NaF, 1:100 Sigma phosphatase inhibitor P2850, 1:100 Sigma protease inhibitor P8340) at a ratio of 1 ml of buffer per 0.1 g of tissue. Homogenates were sonicated twice for 5 s at 3 amperes using a microtip, keeping samples on ice, followed by addition of an equal volume of 2× RIPA buffer. Lysates were incubated on ice for 20 min with periodic mixing by inversion followed by centrifugation at 17,000 × g for 10 min at 4°C. The supernatant was transferred to a fresh tube and quantitated by BCA (Pierce, 23227). Aliquots were flash frozen in liquid nitrogen and stored at −80°C. Twenty μg of whole cell lysate derived from cultured cells or tumors were separated by electrophoresis through Novex 4–12% Tris-Glycine Midi gel (ThermoFisher Scientific, WT4122BOX).

For fractionated lysates from tumors, approximately 0.05 g of frozen tumor tissue was minced and homogenized (no sonication) as described above. Homogenates were centrifuged for 15 min at 600 × g at 4°C. The supernatant was collected as cytosol A and stored on ice. The pellets were resuspended in 0.5 ml of Buffer A1 containing 0.1% Igepal CA-630 and 250 mM sucrose and incubated on ice for 5 min to gently peel off the endoplasmic reticulum membranes. Samples were centrifuged for 10 min at 600 × g at 4°C and the supernatant carefully removed as cytosol B. Cytosols A and B were combined. The pellets representing the nuclei were resuspended in 0.3 ml KCl Extraction Buffer (50 mM Tris pH 7.5, 500 mM KCl, 1:100 Sigma phosphatase inhibitor P2850, 1:100 Sigma protease inhibitor P8340), incubated on ice for 10 min, and sonicated once for 5 s at 3 amperes using a microtip (keeping samples on ice) followed by addition of 0.3 ml of 2× RIPA/Det buffer (2% Igepal CA-630, 1% Na-deoxycholate, 0.2% SDS, 20% glycerol). Samples were incubated on ice for 10 min, centrifuged at 17,000 × g for 10 min at 4°C, and the supernatant transferred to a fresh tube on ice. Lysates were quantitated and aliquoted as described above. Fifteen to 20 μg of each lysate was separated using the NuPAGE 4–12% Bis-Tris Midi gel system (ThermoFisher Scientific, WG1402BOX).

The membranes were processed and antibodies used as described [16, 41]. Antibodies used were: CK2α (A300-197A) and CK2α′ (A300-199A) from Bethyl Laboratories; CK2β (sc-12739 & sc-46666), p-NFκB p65 Ser-529 (sc-101751) and actin (sc-1616) from Santa Cruz Biotechnology; caspase 3 (9662), caveolin-1 (3238), argonaute 1 (5053), argonaute 2 (2897), ERp72 (5033), lamin A/C (2032), NFκB 065 (6956) and Bcl-xL (2762), from Cell Signaling; survivin (AF886) from R&D Systems; GW182 (NBP1-28751) from Novus Biologicals.

Protein signals were quantitated by densitometric analysis using Image J software.

### Quantitative real-time RT-PCR analysis

RNA was isolated and analyzed from frozen cell pellets or tumor tissue as described [16, 17]. FAM TaqMan gene expression probes hs00751002_s1 (CK2α), hs00176505_m1 (CK2α′), hs00365435_m1 (CK2β), and hs01003267_m1 (HPRT-1) were used for analysis of CK2 transcript levels in untreated tumors and cell lines (Table 1). For TaqMan-based analysis of CK2αα′ mRNA levels in nanocapsule treated tumors, primers were designed to specifically amplify the region of the CK2α and α′ mRNAs that was targeted by the RNAi-CK2 and siCK2 oligomers using gene specific forward RNase H-sensitive primers, gene specific Zen-modified FAM labeled internal probes and gene specific reverse primers (Integrated DNA Technologies, Table 6). Conditions included 500 nM primers, 250 nM probe, and 15 mU RNase H (CK2α) or 10 mU RNase H (CK2α′) per 20 μL reaction. PCR was performed on an ABI 7900HT instrument FAST block as follows: 50°C 2 min, 95°C 3 min, 40 cycles of 95°C 10 s and 60°C 30 s. The HPRT-1 gene (Integrated DNA Technologies, hs01003267_m1) was used for normalization.

**Table 6:** PCR Primers

Identifier	Experiment	Sequence^a^
CK2α-LCK-FOR-Gen1-H2	RNAi-CK2 response	TCATGAGCACAGAAAGCTACGAcTAATC^b^
CK2α-LCK-REV-Gen1	RNAi-CK2 response	ATACAACCCAAACTCCACATATCC
CK2α-LCK-Internal	RNAi-CK2 response	TGTCCGAGTTGCTTCCCGATACTT^c^
CK2αp′-LCK-FOR-Gen1-H2	RNAi-CK2 response	GGAGTTTGGGCTGTATGTTAGCaAGCAC^b^
CK2αp′-LCK-REV-Gen1	RNAi-CK2 response	GTACCCAGAACCTTGGCAATG
CK2αp′-LCK-Internal	RNAi-CK2 response	TGGACAGGACAACTATGACCAGCT^c^
LCK-fw	Bioavailability	CGAGATACAACCCAAACTCCA
U6-fw	Bioavailability	CTCGCTTCGGCAGCACA
miRNA-rev	Bioavailability	GCAGGGTCCGAGGTATTC
SLPoly(A)	Bioavailability	GTCGTATCCAGTGCAGGGTCCGAGGTATTCGCACTGG ATACGACAAAAAAAAAAAAAAAAAAVN^d^
SLPoly(A)-LCK	Bioavailability	GTCGTATCCAGTGCAGGGTCCGAGGTATTCGCACTGGA TACGACAAAAAAAAAAAAAAAAAAATGT

a Upper case letters = DNA; lower case letters = RNA.

b 3′ C3 spacer.

c Internal reporter = FAM; Quenchers = Internal ZEN & 3′ Iowa Black FQ.

d V = A, C or G; N = A, C, G or T.

For bioavailability analysis of the TBG-encapsulated oligonucleotide lacking the 3′ propyl modification, the entire tumor, entire spleen and 30% of the liver were pulverized following immersion in liquid nitrogen and RNA was isolated from a 50 mg portion of each pulverized tissue. Following Dounce homogenization in 1.2 ml RLT plus buffer (Qiagen, 1053393), genomic DNA was removed using gDNA Eliminator columns, and the lysate processed for recovery of RNA < 200 nt using the miRneasy kit and RNA mini elute columns as per the manufacturers protocol (Qiagen, 217004). Two 100 mg aliquots of control vehicle treated tumors were homogenized in 2.4 mL of RLT buffer divided into equal parts, spiked with naked RNAi-CK2 3′ unmodified oligomer at either 50 nmol/mg tissue or 25 nmol/mg tumor tissues and RNA < 200 nt isolated as outlined above to determine recovery efficiency of the hybrid DNA/RNA molecule. Standard curves were generated for each plate run using 0.5 μg of total isolated RNA spiked with RNAi-CK2 3′ unmodified oligomer at 2 pmol, 200 fmol, 20 fmol, 2 fmol, and 0.2 fmol. Quantitative stem-loop PCR (q-SL-PCR) was performed as described using the primers listed in Table 6 (Integrated DNA Technologies) using 5 μL of the < 200 nt RNA recovered with the following modifications [42]. Each 20 μL reaction contained a 3:1 mix of the universal and RNAi-CK2i-specific stem-loop primers, and either RNAi-CK2 forward primer or U6 forward primer and the universal reverse primer at the specified concentrations. Reactions were performed on a on an ABI 7900HT instrument FAST block with FAST SYBR green master mix (Applied Biosystems, 4385612) with the following conditions: 95°C 20 s, 40 cycles of 95°C 10 s and 60°C 45 s. PRISM v 6.0 (GraphPad Software) was used for linear regression analysis of the standard curves, which were then used for calculation of the amount of oligomer present the tissues.

### Nanocapsule preparation and characterization

For s50-TBG nanocapsules containing both single- and double-stranded oligomers or erythritol, a dispersion atomization method was used to package the cargo into nanocapsules composed of TBG as described [16, 32]. The sequence for Factor VII (F7) was adapted from a publication [43] and the RNAi-F7 oligomers obtained from TriLink Biotechnologies (San Diego, CA, USA). Physical characterization of particles including average particle size measured by Transmission Electron Microscopy and surface charge determination employed published methods [44].

### Animals

Male athymic nude mice obtained from Frederick National Laboratory (01B474; used for all dose response studies and time course study) and from Charles River Laboratories (Strain Code 490; used for bioavailability study) or NOD-SCID-Gamma (VA internal breeding colony; used for FACS study) were maintained under pathogen-free conditions. Experiments were initiated in nude mice at age 7–8 weeks. NSG mice were used at age 5 months. Mice were housed as described [17]. Facilities at the Minneapolis Veterans Affairs Health Care System were approved by the AAALAC International in accordance with the current regulations and standards of the United States Department of Agriculture (USDA) and National Institutes of Health (NIH) Department of Health and Human Services (DHHS). Animal experimental protocols were approved to be conducted at the Minneapolis Veterans Affairs Health Care System in strict accordance with the recommendations in the Guide for the Care and Use of Laboratory Animals of the National Institutes of Health. The protocols were approved by the Minneapolis Veterans Affairs Health Care System Institutional Animal Care and Use Committee (protocols 131101 and 150201) and by the University of Minnesota Institutional Animal Care and Use Committee (protocol 1312-31154A). All surgical procedures, as approved by the above-mentioned committees, were performed under inhaled isoflurane anesthesia, and all efforts were made to minimize suffering.

### Xenograft tumor initiation, monitoring and treatment

PC3-LN4 and 22Rv1 tumors were initiated by subcutaneous injection of 1 × 10^6^ cells in 50% matrigel (BD Biosciences, 354234) in the mouse flank. Tumor volume was determined every other day by measuring the two longest perpendicular axes with digital calipers and calculated using the formula V = (L × W × W)/2. Therapy was initiated when tumors reached an average size of 80 to 250 mm^3^, depending on the length of time to collect the tumor after initiating treatment. Groups of mice (4 to 9 mice per group as detailed in figure legends) were subjected to intravenous (iv) injection on days 1, 4 and 7 with 0.0001 to 1.0 mg/kg of nanocapsule drug (as detailed per each experiment in Results) diluted in Plasma-Lyte-A (Baxter, 2B2543Q). Mice were sacrificed at time points as described in Results, and tumor, liver, spleen, kidney, and testes were excised, weighed, and snap frozen in liquid nitrogen for protein analysis or placed in formalin. Orthotopic tumors were initiated by injection of LNCaP cells into the prostate and tracked as described [41]. For the bioavailability experiment, mice carrying PC3-LN4 xenograft flank tumors of at least 250 mm^3^ were injected with 5 mg/kg TBG-RNAi-CK2, or 1 mg/kg TBG-RNAi-CK2, or 5 mg/kg “naked” RNAi-CK2 oligomer in combination with TBG-erythritol nanocapsules at the same particle number as the TBG-RNAi-CK2. Groups of 3 mice with tumors of similar volumes (1 per treatment) were injected *via* tail vein and each tumor collected exactly 24 h after treatment.

### Ki-67 and H&E staining of tumors

Immunostaining for Ki-67 and H&E was performed by the Pathology and Laboratory Medicine Service (Minneapolis VA Health Care System). Analysis of the Ki-67 staining was performed using the ImmunoRatio web application [45]. Analysis of the H&E stained tissue sections was performed by visual microscopic inspection by a pathologist of 6 sections per tissue per mouse.

### FACS analysis of xenograft tumors

NOD-SCID-Gamma male mice carrying LNCaP orthotopic xenograft tumors were treated with 200 nmol/kg of TBG-Dy by tail vein or intraperitoneal injection. At 20 h post-injection, the tumors were harvested and processed for FACS analysis of Dy content as described [16].

### Immunofluorescence analyses

Indirect immunofluorescence detection of GW182 (Santa Cruz Biotechnology sc-376939; 1:50) and Syrian hamster IgG were performed essentially as described for FFPE tissue [17].

### Blood serum chemistry analysis

Mice were injected by intraperitoneal route with a lethal amount of Euthasol^®^ solution diluted in normal saline. Once mice were unresponsive to toe pinch, the chest cavity was opened and blood was collected directly from the heart using a 23 gauge blood collection needle (BD, 367342) connected to a 3 ml syringe. The blood was transferred to a minicollect serum separation tube (Greiner Bio-One, 450472), incubated for 30 min at room temperature, and centrifuged 3000 × g for 10 min at room temperature. The serum supernatant was transferred to fresh tubes, flash frozen in liquid nitrogen, and stored at −80°C. Sera were analyzed by the Clinical Pathology Laboratory at the University of Minnesota, College of Veterinary Medicine.

### Statistical analysis

Mean immunoblot protein levels were summarized and compared by treatment group using *t*-tests assuming unequal variance. Means ± standard deviation (SD) are presented. Mouse tumor volumes and primary tumor weights (analyzed on natural log scale) on days 10 or 11 relative to day 0 were summarized and compared by treatment group using analysis of variance (ANOVA). *P*-values for pairwise comparisons for above analyses were conservatively adjusted for multiple comparisons using a Bonferroni correction. The percent Ki-67 positive cells and the serum chemistry values were compared between two treatment groups by *t*-test assuming unequal variances. The comparison of changes in Ki-67 by treatment from day 5 to day 7 was performed using a linear regression model including fixed effects for treatment, day and a treatment by day interaction. *P*-values < 0.05 were considered statistically significant.

### Disclaimer

The views expressed in this article are those of the authors and do not necessarily reflect the position or policy of the U.S. Department of Veterans Affairs or the U.S. government.

## SUPPLEMENTARY MATERIALS FIGURE AND TABLE


