# Supplementary material for: CK2 targeted RNAi therapeutic delivered via malignant cell-directed tenfibgen nanocapsule: dose and molecular mechanisms of response in xenograft prostate tumors
Source: Oncotarget. 2016 Aug 20;7(38):61789–805. doi: 10.18632/oncotarget.11442 (PMC5308691; doi:10.18632/oncotarget.11442)
Supplement: Supplementary file 1 [file oncotarget-07-61789-s001.pdf]

CK2 targeted RNAi therapeutic delivered via malignant cell-directed tenfibgen nanocapsule: dose and molecular mechanisms of response in xenograft prostate tumors

Supplementary Materials

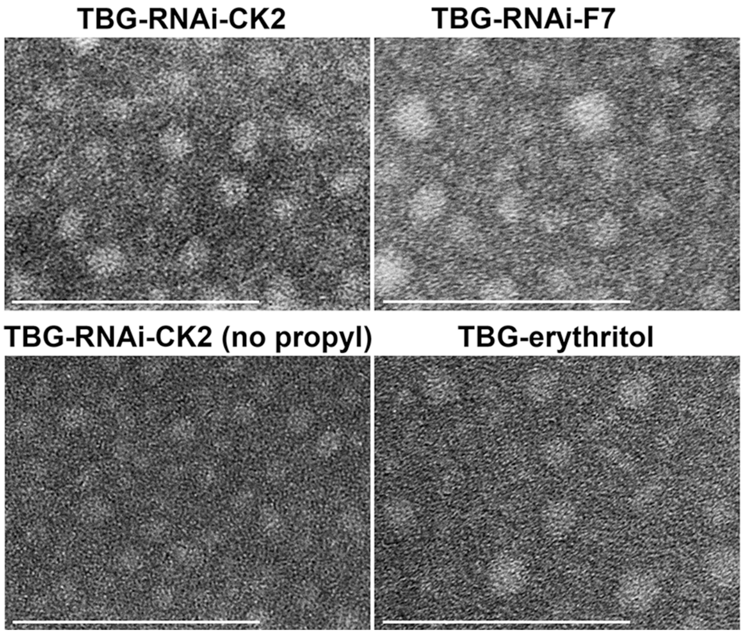

Supplementary Figure S1: Nanocapsule morphology. Transmission electron micrographs of TBG-RNAi-CK2, TBG-RNAi-F7, TBG-RNAi-CK2-no propyl, and TBG-erythritol nanocapsules used for different *in vivo* studies. Scale bar represents 100 nm. Original magnification 80,000.

Supplementary Table S1: TBG nanocapsule characteristics and information

Cargo	Particle Size (nm)[a]	Zeta Potential (meV)[b]	Morphology[c]	Sequence[d]
RNAi-CK2	20.5 ± 2.1	−1.19 ± 1.05	Uniform, Single Capsules	5′ATACAACCCAAACT ccacau(propyl)3′
RNAi-CK2 (no propyl)	18.9 ± 1.7	−12.25 ± 7.4	Uniform, Single Capsules	5′ATACAACCCAAA CTccacau3′
RNAi-F7	21.9 ± 2.3	1.49 ± 0.99	Uniform, Single Capsules	5′GUAAGACTTGAG Augaucc(propyl)3′
Erythritol	22.8 ± 1.9	1.07 ± 0.67	Uniform, Single Capsules	N/A

[a]Mean ± SD of the average elliptical diameter determined from TEM micrographs measuring at least 15 nanocapsules.
[b]Average surface charge measured by dynamic light scattering across a 20 volt potential in 1 mM KCl at 2 µg/ml. Data shown is the mean ± SD of 3 independent measurements.
[c]Morphology of all nanocapsules determined by visual AFM and TEM observation.
[d]Sense strand sequence. Upper case letters represent phosphodiester DNA chemistry; small case letters represent 2′ O-methyl RNA chemistry.
